# What Do You Meme, Professor? An Experiment Using “Memes” in Pharmacy Education

**DOI:** 10.3390/pharmacy8040202

**Published:** 2020-10-29

**Authors:** Joshua D. Brown

**Affiliations:** Department of Pharmaceutical Outcomes and Policy, Center for Drug Evaluation and Safety, University of Florida College of Pharmacy, Gainesville, FL 32610, USA; joshua.brown@ufl.edu

**Keywords:** pharmacy education, modern classroom, memes, social media, digital media

## Abstract

Memes are social or cultural constructs and ideas dispersed from person to person. The modern definition of memes applies to images, photographs, or videos shared on digital platforms juxtaposed with text that utilizes the emotion, meaning, or joke behind the original “meme” to communicate the author’s message. Younger generations of learners are more prone to utilize digital and social media to distribute and consume information and use of these platforms has increased in modern classrooms. However, there are few examples of using memes for educational purposes, including student-generated content, and no known examples in pharmacy education. This commentary introduces the concept of a meme and describes an attempt to incorporate a student-generated meme assignment in a pharmacy course. Experiences and lessons learned are discussed as words of caution in incorporating and evaluating memes or other informal communication tools for educational purposes.

## 1. Generations of Learners

The demographic cohorts of Millennials (born 1981–1996) [1] and Generation Z (born 1997–2012) [2] make up the majority of current students in pharmacy education. These generations are also the next wave of future and current faculty in schools of pharmacy. While many stereotypes are imposed on each generation, consistent and widely accepted characterizations of these cohorts include that they are “digital natives” having been born during or after the advent of the internet and other digital innovations [1,2]. As such, these groups are attributed with higher usage of digital devices, digital media, and social media in their everyday lives including work, play, and school [1,2].

Adapting education to how students learn is important given the contexts of how each generation processes and consumes information [3]. Use of social media, for example, is considered an informal communication approach but facilitates discussion via internet forums such as Reddit or Facebook groups and content creation via platforms such as YouTube or Tik Tok [4]. Within digital and social media are several other styles of non-traditional communication such as the use of “memes”.

## 2. What Is a Meme?

A “meme” has a formal definition not related to our typical connotation of humorous pictures with words. Rather, the definition of a meme is “an idea, behavior, style, or usage that spreads from person to person within a culture” [5]. A modern definition is that memes are “a piece of culture, typically a joke, which gains influence through online transmission” [6,7]. These pieces of information usually take the form of images (pictures; animations such as GIFs) taken from other media (e.g., television or film) or photographs from everyday life, often overlayed with specific phrases or quotes. The intended information extracted from these images or videos is the “meme” which is the underlying message, joke, or emotion the meme represents. Modern memes can usually be described as “tongue in cheek”, irreverent, and humorous, especially dependent on the subject matter they are applied to.

## 3. Recipe of a Meme

**Figure 1:**
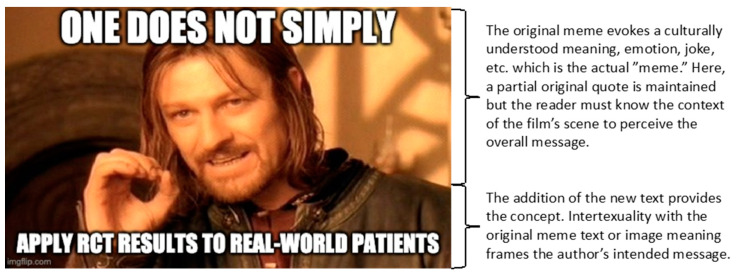
Example of a meme and its components. The original meme is a still image from a popular film. Memes may also be made from photographs, animations, and videos. Though memes often use excerpts from copyrighted material, the edited materials fall under a “fair use” category due to using only a small part of the material. Use for educational purposes and other considerations also define fair use. Created using imgflip.com.

An example of a meme representing a pharmacy course concept is shown in Figure 1. The meme is represented by a still image from *The Fellowship of the Ring*, the first movie and book of the Lord of the Rings trilogy. Actor Sean Bean as the character Boromir states that “One does not simply walk into Mordor”. Culturally, this image has been adapted to have an underlying goal of communicating a task that is difficult or challenging but usually in a humorous manner. Retaining the partial quote “One does not simply…” the added text provides the concept to be communicated by the meme author. Here, specific to the pharmacy course discussed below, the meme communicates that a course concept of “generalizability” is a concern for randomized clinical trials and insinuates that this is a challenge amongst clinicians when treating typical patients in a clinical environment.

Memes can be adapted and re-used in such ways because the underlying message of the meme remains intact and provides context to interpret the new text added by the author. This represents “intertextuality” defined here as when the meaning of text is shaped by the meaning of another text [8]. In memes, this also applies to the implied joke or emotion gleaned from the image used. Successful re-use assumes that the new consumer of this updated meme understands the underlying cultural meaning or interpretation. Of note is that memes stem from an original, potentially copyrighted source and the re-use and adaptation of the original content is defended, in most cases, by “fair use”, thus not in violation of copyright laws [9,10].

## 4. Use of Memes in Education

Use for educational purposes would also be an example of “fair use” of memes. However, there are few examples where memes have been used in education. Wells [11] assigned political science students to create memes paired with an essay to interpret the implied meaning with classmates. Memes have also been used in chemistry test preparation by utilizing memes to link course content to the concept implied by the meme [7]. With few other literature examples, and no examples found specifically in pharmacy education, described below is a personal motivation, experience, and perception of an impromptu, meme-based assignment in a Doctor of Pharmacy (PharmD) curriculum.

## 5. Personal Motivations to Incorporate Memes into the Classroom

As a Millennial, I frequently consume and communicate news, opinions, and other information via memes in both personal and professional interactions. As an educator, I utilize humorous memes to make light of lecture content with the assumption that by creating a memorable image and joke, the concept may be more often recalled by students who may have more visual learning styles. I also create memes in a clear and blatant attempt at connecting with students. In the process of creating these memes, I realized the difficulty involved even for myself, a relative expert in the course subject matter, to create these memes particularly due to trying to adapt the course concept to the meme’s construct.

The course in question is a third-year, PharmD required course on “Pharmacoepidemiology and Drug Safety” at the University of Florida College of Pharmacy. The content covered includes, among others, epidemiological study designs, sources of bias, real-world evidence, comparative effectiveness research, and the roles of the U.S. Food and Drug Administration. Learning objectives for the course are to understand the above concepts and apply them to literature appraisal and discussion of research interpretation and quality. Much of the content is conceptual and new to the students so there is a focus on recall and understanding with some application re-enforced in a final assignment and a future course. That final assignment has traditionally been a full, two-page article critique assessing students’ ability to apply course concepts to a simulated peer-review activity using a previously published article. Through a future course, students are also challenged to adapt course content focused on population-based research to provide personalized patient care. Thus, a core competency expected from the course could be described as the ability to communicate complex studies to a lay person—in this case, a patient.

Given a large focus on conceptual understanding, it seemed obvious that meme creation applied to course content could go beyond typical recall and may even facilitate higher-level thought, application, and retention of these concepts. In addition, given the disruptive events of 2020 and the stress involved, relieving the students of a two-page paper assignment seemed prudent. Thus, as a final course assignment, an assignment to create at least two memes per three-to-four person group was created. Assignment guidelines were simply to apply a course concept to a meme that would be “work appropriate”. The assignment was given and completed during the 2020 Fall semester by approximately 225 third-year pharmacy students.

## 6. Outcomes of the Assignment

Group submissions were informally evaluated for content, correctness, and hilarity. Several examples are available on the instructor’s Twitter feed (@DRxBrown) or available upon request. Direct feedback from students on the assignment was overall positive and appeared enthusiastic with several groups creating more memes than assigned. Additional feedback mirrored the instructor’s experience stating that the application of course concepts to memes was indeed a challenge and generated discussion amongst group members. 

Generated content typically focused on a subset of content that was touched on multiple times throughout the course. These themes included, for example, weaknesses of clinical trials, benefits to conducting real-world studies such as generalizability, use of active comparators, confounding, composite outcomes, and effect modifiers. Content perceived by the instructor as less important was sometimes over-represented by students’ memes. For example, pragmatic clinical trials were mentioned in approximately one in five of the submitted memes, although this type of research was discussed but not intended as a point of emphasis in the course.

Overall, five general themes evolved from the nature of the memes submitted, which should be considered further by instructors providing such assignments:

1. Students provided clear application of course concepts, demonstrating critical thinking and communication through the meme.
2. Students re-enforced a teaching point such as focusing on a prior missed or particularly tricky quiz question or a key discussion point during lectures.
3. Students incorrectly attributed a concept to the meme or used incorrect concept terminology when making the meme.
4. Via themes one through three above, patterns emerged showing potential deficiencies in lecture materials that may necessitate future course updates, including concepts that may be over- or under-emphasized in lectures and other course activities.
5. Students created memes about tangential topics or course events. For example, students focused memes on quotes from the instructor without a direct application to the course content.

Arguably, themes one through three should be viewed positively as they demonstrate opportunities for students to learn the content through the assignment and direct feedback from the instructor. Theme four suggested course content that may need to be bolstered or reduced as students focused too often on a certain concept or repeatedly misrepresented a concept over multiple group submissions. Theme five also suggested that students may require additional guidance and refinement of the assignment to focus on course concepts.

## 7. Instructor’s Post-Assignment Reflection

While not formally evaluated, this meme assignment displayed an overall ability of PharmD students to apply challenging course concepts to create new memes. Moreover, this assignment was positively accepted by students and feedback suggested that the activity supported an active learning environment amongst group members. Multiple students also indicated uncertainty about the assignment initially but acknowledged that the task of transferring course content to a meme facilitated discussion amongst group members. In most cases, students aptly applied course concepts, although some clarification was made when needed and this provided an additional teaching opportunity. The activity was also perceived to have revealed opportunities for the instructor to continuously improve course content. In addition, it provided the instructor an opportunity to individually engage with groups within a large (>200) student body spread over three campuses where this may have not been otherwise possible. I believe it was perceived as a humanizing task where humor was shared in addition to pictures of pets, background stories to the memes (e.g., “this is from my favorite movie”). In a time of increased virtual learning, overlaying these opportunities to connect and share appeared to be particularly appreciated by students (and the instructor!).

Sharing the activity via Twitter also provided an opportunity to gauge feedback from peer colleagues within—and outside—the university. Among fellow researchers and educators on Twitter, feedback was overwhelmingly positive and total engagements of >10,000 were noted as was individual feedback showing personal feelings towards concepts or topics addressed by individual memes. Feedback from this group indicated clear educational value and some educators have already engaged this activity in coursework at other colleges of pharmacy. One challenge, which is noted in more detail in the following paragraph, is communicating the activity outside of an environment like Twitter where users are clearly social media oriented. Sharing the activity with colleagues that may be classified as “Gen X” or older and less prone to such communication media, was met with some uncertainty towards the educational value and overall concept of a “meme”. While not experienced here, it could also be a challenge to argue about the professional aspects of this activity in a formal learning environment especially among colleagues with stricter rules about what constitutes “professionalism”.

Use of memes in higher education may fit into the “21st Century Learning Environment” but the experience described here and elsewhere warrant some points of caution that are perhaps germane to many innovative teaching approaches [3]. For one, the onus is on instructors to “keep up” by staying current on emerging trends of meme use in order to effectively communicate with students. Emphasizing a generational gap between professor and student by incorrectly attributing meaning to memes may mitigate the desired effects of connection and learning in the same way that references to “out-of-date” or “old school” musicians or television shows often do [3]. In addition to misinterpretation, humor and emotions are typically subjective, and some leniency must be considered if interpretation is part of the assessment. It has also been suggested that the relative knowledge level of the course content should be considered prior to engaging in informal learning activities [3]. In this case, with third-year PharmD students who have been exposed to many of the concepts and have extensive clinical knowledge, it was deemed appropriate. However, in cases where content is new and foreign to students, this may be a daunting task, for example, in first-year students who may be adapting to not only new content but also a new degree program.

Moving forward, formal educational evaluation is needed to investigate the potential of incorporating memes into higher education curricula, including at the professional and graduate levels. In particular, evaluating what knowledge and skills memes represent will be an important exercise. While recall and application learning taxonomies may be apparent, content creation may also be a practical way for these learners to connect to patients and the public within their own generations. Given the divides in communicating science to the public, in the same way we should adapt to our learners’ learning styles, our graduates may also need to adapt to better communicate and disseminate scientific and clinical knowledge. By incorporating such informal activities in a professional learning environment, we may even create a safe space to develop good practices in these types of communications and create a generation of professionals comfortable with both formal and informal means of communicating science and health information.

## 8. Conclusions

Use of memes, digital technology and media, and social media are the common tools of communication among Millennials and Generation Z learners. Integrating these into courses may blur the line between formal and informal, professional and unprofessional, communication but could help bridge generational gaps. More importantly, it engages the learner using the format they often use to consume information. Use of memes deserves further evaluation of its impact on learning outcomes. Prior to use, instructors should consider the level of knowledge needed to apply difficult concepts in this format and the instructor’s own ability to interpret and present the activities being requested of students.
